# Behavior and morphology combine to influence energy dissipation in mantis shrimp (Stomatopoda)

**DOI:** 10.1242/jeb.247063

**Published:** 2024-05-09

**Authors:** P. A. Green

**Affiliations:** ^1^ UC Santa Barbara, Ecology, Evolution, and Marine Biology, Santa Barbara, CA 93106, USA; ^2^ Brown University, Ecology, Evolution, and Organismal Biology, Providence, RI 02912, USA

**Keywords:** Biomechanics, Animal behavior, Coefficient of restitution, Animal contests, Ecomorphology

## Abstract

Animals deliver and withstand physical impacts in diverse behavioral contexts, from competing rams clashing their antlers together to archerfish impacting prey with jets of water. Though the ability of animals to withstand impact has generally been studied by focusing on morphology, behaviors may also influence impact resistance. Mantis shrimp exchange high-force strikes on each other's coiled, armored telsons (tailplates) during contests over territory. Prior work has shown that telson morphology has high impact resistance. I hypothesized that the behavior of coiling the telson also contributes to impact energy dissipation. By measuring impact dynamics from high-speed videos of strikes exchanged during contests between freely moving animals, I found that approximately 20% more impact energy was dissipated by the telson as compared with findings from a prior study that focused solely on morphology. This increase is likely due to behavior: because the telson is lifted off the substrate, the entire body flexes after contact, dissipating more energy than exoskeletal morphology does on its own. While variation in the degree of telson coil did not affect energy dissipation, proportionally more energy was dissipated from higher velocity strikes and from strikes from more massive appendages. Overall, these findings show that analysis of both behavior and morphology is crucial to understanding impact resistance, and suggest future research on the evolution of structure and function under the selective pressure of biological impacts.

## Teaser

**Highlighted Article:** Freely competing mantis shrimp dissipate 90% of the energy of high-force strikes by raising their impact-resistant tailplates off the substrate; faster strikes led to greater proportional energy dissipation.

## INTRODUCTION

Animals deliver and sustain physical impacts across contexts, from capturing prey (Vailati et al., 2012) to escaping predators (Patek et al., 2006). Impacts may be particularly prevalent in contests, where taxa as diverse as rams (Geist, 1966), parrotfish (Muñoz et al., 2012) and hermit crabs (Briffa and Elwood, 2001) collide body parts together in fights. The ability to dissipate impact energy may determine the risk of injury an individual faces in a contest, and thereby affect contest success. Further, contest success dictates whether or not an individual can access resources that are essential to fitness, such as mates and food. Because of the links between impacts, contest success and fitness, the ability to dissipate impact energy during contests may exert a selective force on animal morphology and behavior, selecting for materials, structures and behaviors that help dissipate impact energy.

A significant body of literature has addressed how materials and structures (hereafter referred to as ‘morphology’, *sensu* Garland and Losos, 1994) withstand impacts. For example, techniques such as finite element analysis (Drake et al., 2016; Johnson et al., 2021) and manipulative experiments (Kingston et al., 2022) have shown how bone and cuticle structures dissipate impact energy to protect animal brains. Other approaches have shown the importance of material properties to impact energy flow. For instance, trap-jaw ants close their jaws at ultrafast speeds, using these impacts for multiple purposes, from capturing soft-bodied prey to escaping predation by propelling themselves off hard substrates (Spagna et al., 2009). By studying the kinematics of trap-jaw ant impacts, Jorge et al. (2021) found that jaw closure transferred more energy to stiff targets than to compliant ones, showing how the same impact, delivered to different materials, can achieve different biological tasks.

Impact energy dynamics may also be affected by behavior; that is, how animals use their morphology (*sensu* Garland and Losos, 1994). For example, hermit crabs compete over the shells they use to protect their bodies. During contests, one individual displaces its shell away from that of its opponent, then moves its shell back to collide with the opponent's shell (Elwood and Briffa, 2001). These ‘attackers’ vary in where they land impacts on the shell of a ‘defender’ (Lane and Briffa, 2020) and the distance they displace their shell before starting their impact motion (Briffa and Fortescue, 2017), behaviors that could affect how impacts are received by opponents. Further, this behavioral variation can affect contest success: compared with losers of hermit crab contests, winners were more likely to land their impacts on a specific portion of the defender's shell (Lane and Briffa, 2020), and to displace their shells a shorter distance away from the defender's shell (Briffa and Fortescue, 2017). Combined, these examples in hermit crabs show how behavior may affect impacts and, therefore, contest success. However, despite work in organismal biology that has emphasized the importance of both morphology and behavior to organismal performance (Garland and Losos, 1994; Green et al., 2021; Lauder, 1995), little research has taken this integrative approach to understand impact energy dissipation in animal systems.

Here, I used the coefficient of restitution (COR), a common engineering metric that quantifies the relative velocity of objects before and after their collision (Hibbeler, 1992), to understand the combined importance of behavior and morphology to impact energy dissipation. COR usually ranges from zero to one: a COR of zero represents a fully plastic impact, in which all impact energy is dissipated and the two colliding objects stick together, whereas a COR of one represents a fully elastic impact, such that no energy is lost and the objects bounce off in opposite directions with the same velocity as before their collision. Though COR is sometimes reported as a measure of a material (e.g. ‘the COR of a baseball bat is X’), it is more accurately a measure of a given impact (e.g. ‘the COR of a baseball bat hitting an 80 mph curveball is X’), and factors such as the velocity and angle of impact can affect COR (Andersen et al., 1999; Chau et al., 2002; Haron and Ismail, 2012; Hibbeler, 1992). Because COR combines aspects of both morphology (e.g. shape and material properties) and behavior (e.g. impact velocity), it can be a useful metric for understanding the contributions of these factors to impacts in animal systems.

I studied COR in mantis shrimp, an influential system in the study of biological impact (reviewed in deVries et al., 2021; Patek, 2019). During contests over burrow territories, mantis shrimp repeatedly deliver high-force impacts from raptorial appendages onto each other's coiled telsons (tailplates) in a behavior termed ‘telson sparring’ (Green and Patek, 2015). Both the appendage (Weaver et al., 2012) and telson (Yaraghi et al., 2019; Zhang et al., 2016) have morphological properties that help them withstand impacts, including increased mineralization at areas where contact occurs (Taylor and Patek, 2010). Following ‘ball drop’ methods common in the engineering literature, Taylor and Patek (2010) measured the COR of telson exoskeleton in the mantis shrimp species *Neogonodacylus wennerae* by dropping a steel ball onto a telson fixed to a lab bench and comparing the ball's velocity as it rebounded off the telson (i.e. post-impact) with the ball's velocity as it fell (i.e. pre-impact). They found that the telson dissipated, on average, approximately 69% of the energy of an impacting strike (Taylor and Patek, 2010). Further, COR varied with the body mass of the tested individual – the exoskeletons of larger shrimp dissipated proportionally more energy than those of smaller shrimp (Taylor and Patek, 2010).

**Fig. 1.**
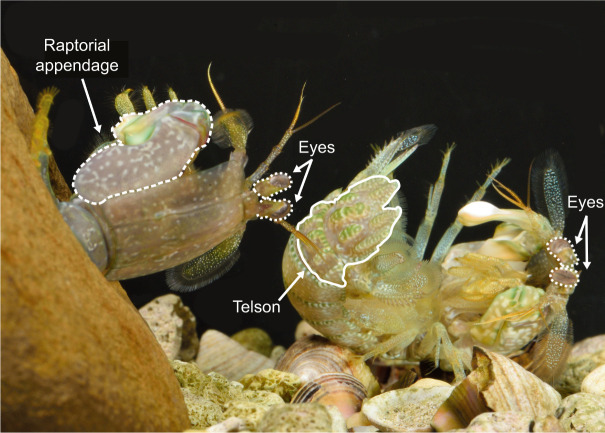
**During contests, mantis shrimp lift their telsons off the substrate in a ‘telson coil’ behavior.** Here, the individual on the right is coiling its telson (outlined in solid white) while lying on its left side (dorsal into page). The individual on the right is exiting a burrow (dorsal out of page), with its raptorial appendage outlined in dashed white. The eyes are outlined in dotted white lines. Photo: Roy Caldwell.

In addition to telson morphology, how mantis shrimp use their telsons may affect impact energy dissipation during telson sparring. During natural interactions, instead of laying their telsons on the substrate, competitors raise their telsons in front of their bodies in a ‘telson coil’ behavior (Fig. 1). The ability of the coiled telson to flex with the rest of the animal's body after impact – much like a boxer moves with a punch they receive – might increase energy dissipation (see also Taylor and Patek, 2010). Furthermore, striking individuals can vary the angle and velocity with which they strike in ways that may change energy dissipation. Green and colleagues (2019) found that mantis shrimp fighting against relatively smaller competitors delivered lower-velocity, lower-energy strikes than those fighting against relatively larger competitors. That is, competitors changed their striking behavior according to their opponent’s size. Variation in impact velocity (Asteriou and Tsiambaos, 2018; Haron and Ismail, 2012) has been shown to lead to changes in energy dissipation in the engineering and sports science literature. Overall, in addition to morphological variation, behavioral variation might affect the COR of telson sparring impacts.

To understand the importance of both morphology and behavior on impact energy dissipation in mantis shrimp, I measured COR from live, sparring competitors. I first asked whether and how my measures of COR that incorporated both morphology and behavior differed from those that focused only on morphology (Taylor and Patek, 2010; Taylor et al., 2019). I hypothesized that, because the telson and the rest of the body can move freely via the telson coil behavior, the COR of impacts from live competitors would be lower (i.e. more energy dissipated) than that measured by Taylor and Patek (2010). I also asked whether variation in behavior or morphology that is both common to other studies of COR (impact velocity, object mass; e.g. Chau et al., 2002; Cross, 2002; Haron and Ismail, 2012) and unique to the mantis shrimp system (angle of the telson coil, angle of the appendage at contact) affected variation in COR. Following the engineering literature, I hypothesized that greater appendage mass of striking individuals and greater impact velocity would result in decreased COR (Asteriou and Tsiambaos, 2018; Chau et al., 2002; Haron and Ismail, 2012). I also hypothesized that, if the telson coil behavior has evolved (at least in part) to facilitate impact energy dissipation, increases in the degree to which the telson was coiled would result in lower COR. Finally, I tested the effect of the angle of the appendage at contact (compared with that before the strike motion) on COR. Though this factor has not been specifically studied in terms of its effect on properties such as strike force, I noticed substantial variation in this angle across studied strikes (minimum angle at contact=17.94 deg, maximum angle=88.20 deg) that I hypothesized might change how an impact is delivered to a competitor's telson, e.g. the force delivered or the directionality of that force. I did not have an *a priori* directional hypothesis as to how appendage angle at contact would affect COR.

## MATERIALS AND METHODS

The contests studied here were originally studied in Green et al. (2019).

### Animal collection and care, and contest staging

I collected *Neogonodactylus bredini* (Manning 1969) from burrows in coral rubble in Panama (Autoridad Nacional del Ambiente collection permits SE/A-115-13, SE/A-92-15, SE/A-52-17), transported them to Duke University, and housed them individually in 10 cm^3^ plastic cubes in an aquarium with circulating artificial sea water (27°C, 12 h:12 h light:dark cycle). Each individual was provided with a burrow refuge made of PVC tubing cut longitudinally and secured to the side of the cube, and was fed twice weekly with frozen krill and brine shrimp, or fresh snails.

I staged contests between competitors that were matched for body size within the range of prior studies (Green and Patek, 2015; Green and Patek, 2018; mean±s.d. body mass 2.00±0.40 g, range 0.47–4.60 g). Measurements of body mass and body length were taken after contests using a digital scale (Denver Instruments APX-3202 balance, Sartorius AG, Göttingen, Germany) and digital calipers (Mitutoyo Digimatic Caliper, Mitutoyo Corp., Kawasaki, Japan).

Contests were staged by introducing a competitor in a PVC burrow in front of the burrow of a second individual. After initially separating competitors with an opaque barrier, I removed the barrier and filmed sparring strikes with 30,000 frames s^−1^ (15 strikes) or 40,000 frames s^−1^ (13 strikes) high-speed video (Photron SA-Z or SA-X2 camera; 10–15 μs shutter duration; pixel resolution: 1024×512 or 688, SA-Z; 896×496, SA-X2; Photron FastCam Viewer v3; Photron, San Diego, CA, USA). Competitors readily sparred after the barrier was removed; fighting behaviors were similar to those observed in previously studied interactions (Green and Patek, 2018). I recorded up to three strikes before memory limits in the high-speed video camera required me to stop filming to save the videos to an external hard drive. While saving videos, I replaced the barrier to prevent competitors from further sparring. Once videos were saved, I removed the barrier again to allow competitors to continue sparring. I used either competitor in up to three total ‘bouts’ (i.e. the first contest and then up to two re-introductions) and then did not use either competitor in any other trial that day. Competitive behaviors were consistent across time for all competitors; there were no indications of fatigue during testing. Random effects of individual identity were used in all statistical models to control for repeated measures from each individual (see below). Summary statistics are reported in the Results.

### Quantifying appendage and telson motion, and COR

I first discarded any high-speed videos where I could not fully quantify appendage motion or where the appendage or telson moved off-axis (i.e. moved out of focus) within five frames immediately before or after the strike motion. Prior work has shown that mantis shrimp strikes that remain in focus for the entire duration of digitized movement have minimal digitizing error (DeVries et al., 2012).

**Fig. 2.**
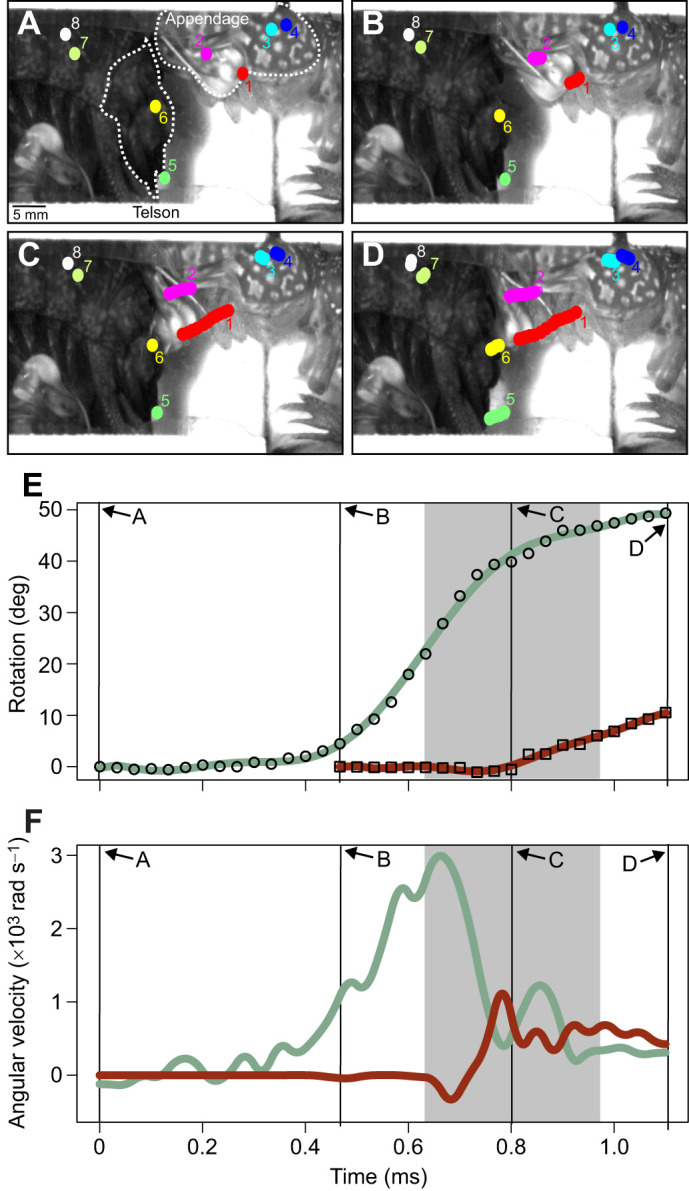
**High-speed videos of strikes were used to quantify appendage and telson rotational displacement and velocity, and to calculate the coefficient of restitution (COR).** (A–D) Still images from high-speed video showing points digitized on the striking body (1, 2) and merus (3, 4) of the striking individual, and on the telson (5, 6) and abdomen (7, 8) of the individual receiving the strike. Appendage and telson are outlined with dotted white lines and indicated in A; the individual receiving the strike has the appendage at the bottom. (E) Plot of rotational displacement (*y*-axis) over time (*x*-axis) of raw data (points) and polynomial fit (lines) of appendage (open circles, green line) and telson (open squares, red line). (F) Plot of angular velocity (*y*-axis) over time (*x*-axis) of appendage (green line) and telson (red line). In both E and F, solid vertical lines correspond to images in A–D; line at 0.8 ms is frame of contact. Shaded gray area indicates five frames before and after frame of contact, the range over which angular velocity (slope of displacement data) before and after contact was averaged.

From high-speed videos, I digitized the movement of the appendage and the telson using landmarks or natural brightness patterns, following methods in Kagaya and Patek (2016) and using the MtrackJ plugin in Fiji v.1.53 (Meijering et al., 2012; Schindelin et al., 2012). I digitized appendage movement from the beginning of the strike motion until 10 frames after it made contact with the telson, and telson movement from 10 frames before the appendage made contact until 10 frames after contact (Fig. 2). Note that, in some cases, contact occurred between two frames; in these cases, I chose the latter frame as the frame of contact, to ensure I was identifying when contact occurred and not before.

From the digitized points, I generated rotational displacement data (Fig. 2E) using R (http://www.R-project.org/) code originally published by Kagaya and Patek (2016; Fig. 2E). I then used MatLab (R2023a) code developed in prior work (Green et al., 2019; McHenry et al., 2012, 2016) to calculate appendage and telson angular velocity (Fig. 2F). This code fits a 7th–9th order polynomial spline (spline order evaluated visually for each strike) to the raw displacement data, interpolates 5000 points along the spline, and takes the derivative of displacement with respect to time to calculate velocity.

From angular velocity data, I calculated COR using two methods. The first method replicates the ball-drop test used by Taylor and Patek (2010; see also Taylor et al., 2019) by assuming the telson acts as an immobile surface. This method calculates COR as:
(1)

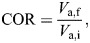
where *V*_a,f_ is the mean angular velocity of the appendage over five high-speed video frames immediately after contact and *V*­_a,i_ is the mean angular velocity of the appendage over five frames immediately before contact (gray regions in Fig. 2E,F). While, in the live interactions I filmed, the appendage often moved beyond the point of contact with the telson (i.e. continued moving in the same direction as pre-contact, see Results), in the ball-drop tests used by Taylor and Patek (2010), the colliding object could only rebound, not continue its motion, as the telson was secured to a solid lab bench. To allow for as much of a direct comparison as possible to these prior measurements, in my calculations of COR using Eqn 1, I ignored the directionality of appendage motion.

The second method treats the telson as a second particle, similar to two balls colliding, and accounts for the telson's movement before and after contact (Hibbeler, 1992):
(2)

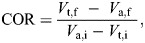
where *V*_t,f_ and *V*_a,f_ represent telson and appendage angular velocity over five frames immediately after contact, respectively, and *V*_t,i_ and *V*_a,i_ represent telson and appendage angular velocity over five frames immediately before contact, respectively (gray regions in Fig. 2E,F). Following Hibbeler (1992), in Eqn 2, I specified the direction of velocity, such that movement of the appendage toward the telson (or telson motion in the same direction) was treated as positive.

In some strikes (e.g. Fig. 2), telson velocity increased before the appendage made contact. This likely occurred because mantis shrimp often (20/28 strikes) struck with both of their raptorial appendages, and at times the appendage out of view of the camera (i.e. the untracked appendage) contacted the telson before the tracked appendage did. Whether or not the untracked appendage made contact before the tracked appendage did not affect my COR measurements using either Eqn 1 [linear mixed model (LMM) of effect of both appendages striking on COR; β=−0.11, s.e.=0.09, χ^2^=1.63, *P*=0.20] or Eqn 2 (β=−0.11, s.e.=0.14, χ^2^=0.65, *P*=0.42).

Importantly, my calculations of COR used the angular velocity of the appendage and telson (measuring their rotation) instead of their linear velocity (measuring translation). While rotational measures are common in and appropriate for this system (e.g. DeVries et al., 2012; Green et al., 2019; Harrison et al., 2021), I also calculated COR using the linear velocity of the appendage and the telson. Here, I multiplied the average rotational velocity of the appendage or telson measured over five frames immediately before or after contact by the length of the striking body (for appendage velocity, the length of the dactyl and propodus) or the length of the telson (for telson velocity; Harrison et al., 2021). Striking body length was measured using digital calipers. Because telson length was not measured at the initial time of the experiment, I measured telson length using the measurement tool in Fiji (v.1.53). I first measured the distance in pixels from the distal tip of the telson to its joint with the next most anterior body segment, then calculated the distance in millimeters by calibrating this pixel measurement with a millimeter-scale ruler placed in the high-speed video frame. In five videos, there was no ruler in the video frame; in these cases, I calibrated the pixel length of the telson by the known millimeter length of the striking individual's striking body. The results did not differ when using either rotational or linear velocity to measure COR (see Tables S1–S3). Prior research has used angular values to calculate COR (Hlosta et al., 2018). Therefore, I report COR as calculated by angular velocity in the main text, and further use of the term ‘velocity’ refers to angular velocity unless otherwise noted.

### Morphological and behavioral correlates of COR

**Fig. 3.**
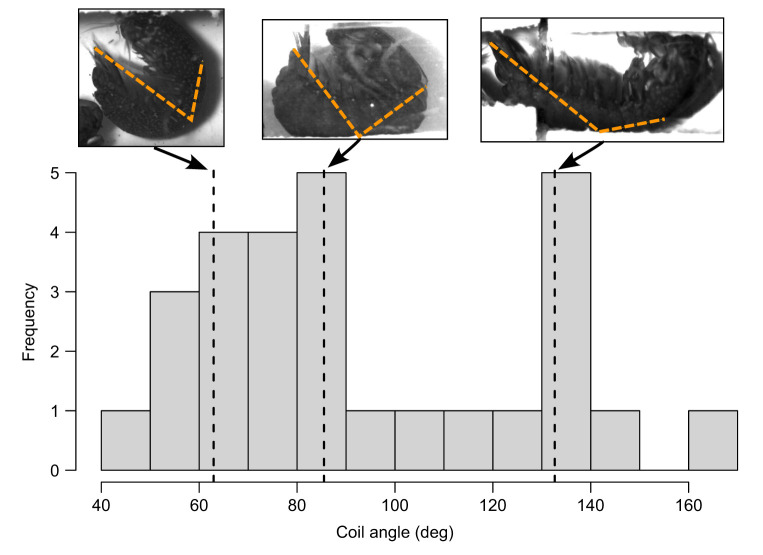
**Histogram of variation in telson coil angle.** Inset images above the histogram (corresponding to the dashed vertical lines) show examples of contact angle variation. Dashed orange lines in the inset images show the lines used to calculate coil angle. In all example images, the dorsal side of the mantis shrimp faces down.

I quantified hypothesized morphological and behavioral correlates of COR (see Introduction). These included (1) the angle of appendage displacement at the time of contact; (2) the velocity of the strike at the time of contact; (3) the angle of the telson coil adopted by the individual receiving the strike; (4) the mass of the moving segments (dactyl and propodus) of the striking individual's appendage, termed ‘striking body mass’ (see also Green et al., 2019); and (5) whether or not the strike was a glancing blow. Metrics 1 and 2, the angle and velocity of the appendage at contact, were quantified through the MatLab code described above. Metric 3, the angle of the telson coil, was quantified as the interior angle of an open triangle drawn (in Fiji v.1.53) from the tip of the telson, to the distal point of the 5th abdominal tergite (approximately halfway between the telson and carapace), to the distal edge of the carapace; lower values represented an individual that adopted a more coiled posture (Fig. 3 and Movie 1). Metric 4, the mass of the striking body, was quantified using a scaling relationship between striking body length and mass developed by Green et al. (2019). Metric 5 was a binary (yes/no) variable that was quantified by observing the high-speed videos and noting when the appendage made contact and then seemed to ‘slide’ or ‘glance’ off the telson surface (see examples in Movie 1).

### Statistical analyses

All statistical analyses were conducted in R v.4.2.1 (http://www.R-project.org/).

I first compared measurements of COR with the raw data of Taylor and Patek (2010), found in the supporting data of Taylor et al. (2019). These data were collected from *Neogonodactylus wennerae*, whereas I filmed interactions between *N. bredini*. However, these species are closely related (Taylor et al., 2019): both Caribbean and visually indistinguishable, including in telson morphology (P.A.G., personal observation). Further, measures of COR from a limited sample (*N*=5 individuals) of *N. bredini* are within the range of the larger sample size (*N*=16) of *N. wennerae* used in Taylor et al. (2019).

I built a LMM (lme4 package; Bates et al., 2015) predicting COR from data type: data from Taylor and Patek (2010), data from the present study using Eqn 1, and data from the present study using Eqn 2. This model also included random effects of the identity of both competitors to account for individuals producing multiple strikes and taking part in multiple contests. After checking a histogram of model residuals to ensure normality, I tested for the significance of data type using the Anova function in the car package (Fox and Weisberg, 2019), and conduced Tukey *post hoc* tests of two-way comparisons using the emmeans function in the emmeans package (https://CRAN.R-project.org/package=emmeans).

To test what behavioral and morphological variables predicted variation in COR, I built two LMMs predicting COR as measured by Eqn 1 and, separately, Eqn 2. Predictor variables were those described above. I also included an interaction between strike velocity at contact and striking body mass. I initially included an interaction between appendage angle at contact and coil angle, but removed this because of high variance inflation factors (Zuur and Ieno, 2016; Zuur et al., 2010). Though COR as measured by Eqn. 1 does not account for appendage direction, following initial plotting I included in this model a predictor variable of appendage movement post-contact, as a two-level factor differentiating whether the appendage moved back toward the striking individual (‘rebound’) or continued to move beyond the point of contact (‘continue’). Finally, I included random effects of the identity of both competitors. I checked histograms of model residuals to ensure good model fit and tested for significance on the full model using the Anova function in the car package.

## RESULTS

I gathered data on 28 strikes from 12 contests between 10 unique striking individuals and 11 unique individuals receiving strikes. The number of strikes analyzed per contest ranged from one to six. I gathered data on COR from appendage movement only (Eqn 1) on all 28 strikes, and from appendage and telson movement (Eqn 2) from 19 of these strikes. In nine cases, I could not digitize telson movement because of blocking of landmarks by cavitation bubbles or other obstructions.

**Fig. 4.**
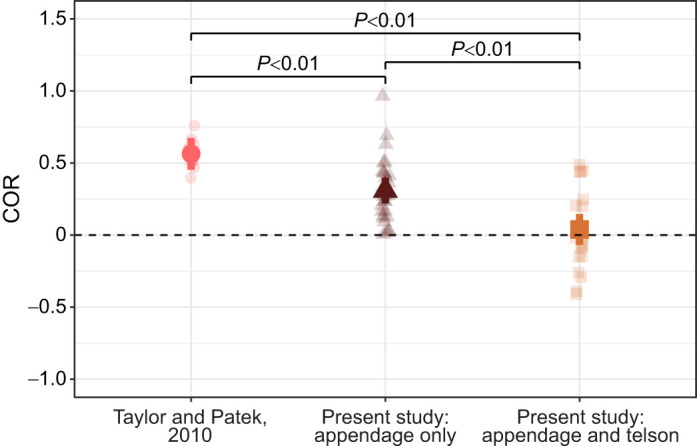
**COR was lower when incorporating both behavior and morphology.** Data from Taylor and Patek (2010) represent morphology only (pink circles); data from the present study measured appendage movement only (brown triangles) and both appendage and telson movement (orange squares). Semi-transparent points represent raw data. Solid points and error bars show estimates and 95% confidence intervals from the model described in Materials and Methods; *P*-values from the model are indicated. Sample sizes were: *N*=16 strikes (Taylor and Patek, 2010); *N*=28 strikes, 10 striking individuals, 11 individuals receiving strikes (present study, appendage movement only); *N*=19 strikes, 9 striking individuals, 10 individuals receiving strikes (present study, appendage and telson movement). Horizontal dashed line shows COR of zero.

Measures of COR from live sparring interactions were significantly lower, indicating more energy was dissipated, than those that measured morphology only (Fig. 4, Table 1). Incorporating both appendage and telson motion (Eqn 2) resulted in lower COR than that from measures of just appendage motion (Eqn 1; Fig. 4, Table 1). Notably, negative COR values often occurred from measures incorporating both appendage and telson motion (Fig. 4). These values occurred because the appendage moved faster than the telson after contact (e.g. Fig. 2; Fig. S1). In these cases, the numerator of Eqn 2 (post-contact motion) was negative, whereas the denominator was always positive, as the appendage always moved faster than the telson pre-contact.

**Table 1.**
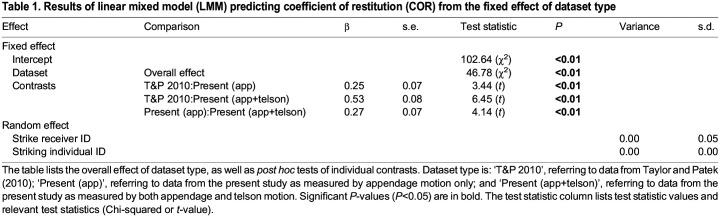
Results of linear mixed model (LMM) predicting coefficient of restitution (COR) from the fixed effect of dataset type

**Fig. 5.**
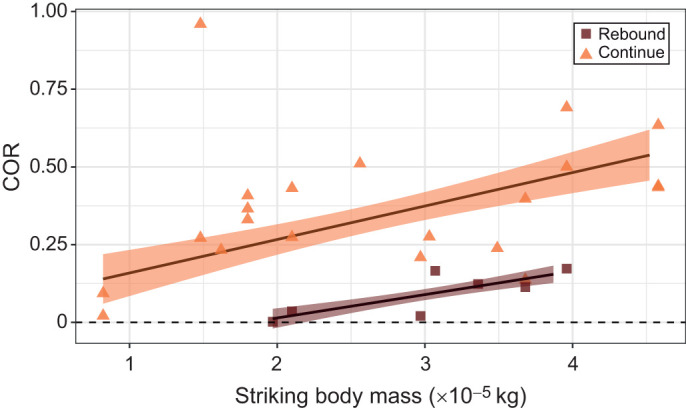
**COR as measured by appendage motion only increased with increasing striking body mass.** Strikes in which the appendage rebounded after contact (brown squares) had an overall lower COR than strikes in which the appendage continued in its initial direction post-contact (orange triangles). Points represent raw data. Solid lines and ribbons represent estimates and s.e., respectively, from models described in Materials and Methods. Because of the small sample size of strikes in the ‘rebound’ category, the estimate line and ribbon are from a simple linear model predicting COR from striking body mass and are meant for visualization purposes only. Sample size was *N*=27 strikes, 11 striking individuals, 10 individuals receiving strikes. Horizontal dashed line shows COR of zero.

When measuring appendage motion only (Eqn 1), strikes from appendages with greater striking body masses resulted in higher COR (Fig. 5, Table 2). Additionally, strikes in which the appendage ‘rebounded’, i.e. moved back toward the striking individual after contact, had lower COR than strikes in which the appendage continued to move in its initial direction after contact.

**Table 2.**
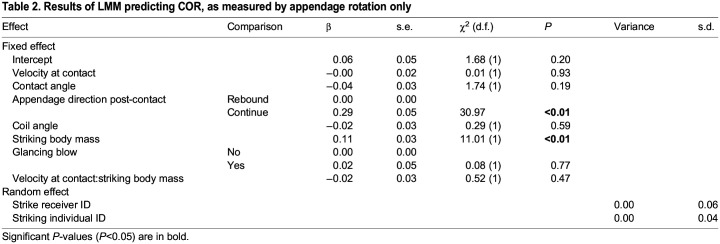
Results of LMM predicting COR, as measured by appendage rotation only

**Fig. 6.**
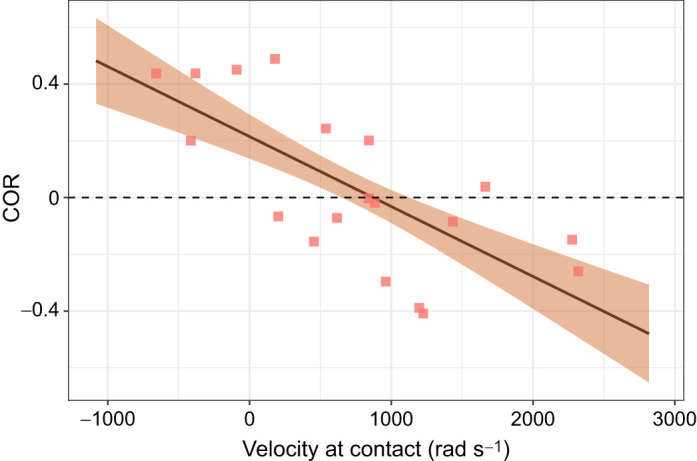
**COR for appendage and telson data decreased with increasing velocity at contact.** Points represent raw data. Solid line and ribbon represent estimate and s.e., respectively, from models described in Materials and Methods. Sample size was *N*=19 strikes, 9 striking individuals, 11 individuals receiving strikes. Horizontal dashed line shows COR of zero.

When measuring appendage and telson motion (Eqn 2), strikes with higher contact velocities resulted in lower COR (Fig. 6, Table 3). Four strikes had a negative velocity at contact (negative *x*-axis values in Fig. 6); in these cases, contact occurred slightly before the frame of contact but after the frame prior. A re-analysis setting the frame of contact in these strikes to one frame prior to the previously designated contact frame, and thereby making contact velocity positive, still showed a negative relationship between contact velocity and COR, but this effect was not statistically significant at the *P*<0.05 level (Table S4).

**Table 3.**
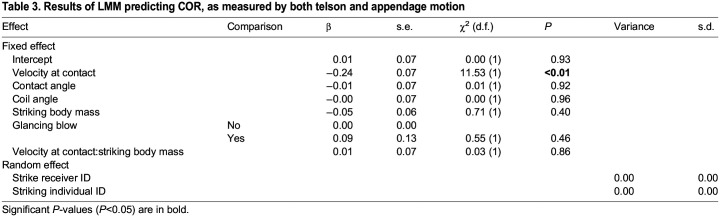
Results of LMM predicting COR, as measured by both telson and appendage motion

## DISCUSSION

Measurements from freely interacting competitors reveal the contributions of both morphology and behavior to energy dissipation in animal impacts. Previous work using a ball-drop technique that focused only on the morphology of the telson exoskeleton found it dissipates approximately 69% of impact energy (energy dissipation calculated as 1−COR^2^; Taylor and Patek, 2010). When comparing my data using the same equation as that used in a ball-drop test (Eqn 1), I found mean energy dissipation of approximately 90%. This difference is most likely due to the telson coil; that is, the behavior with which competitors use their telson. By lifting the telson off the substrate, the whole body of the animal flexes upon receiving a strike (Movie 1), much like a boxer moving with a punch they receive. As a result, energy is dissipated throughout the body and not just in the telson exoskeleton, an effect that Taylor and Patek (2010) suggested in their study. Importantly, my results do not suggest those of Taylor and Patek (2010) are inaccurate. Rather, they add information to this prior study of morphology by showing how the behavior of the telson coil can affect energy dissipation. Other factors may also increase energy dissipation, including loss of energy to cavitation (Patek and Caldwell, 2005). I observed cavitation upon contact in every strike I recorded, and at times cavitation bubbles developed far beyond the location of the strike (e.g. on the ventral side of the telson or proximally along the abdomen).

Although COR is usually between 0 and 1 (Hibbeler, 1992), COR when incorporating both appendage and telson motion (Eqn 2) was often negative (Fig. 4). Negative COR values have also been described in engineering (Müller et al., 2012) and sports science (Cross, 2002), especially for oblique impacts like those studied here. As described in the Results, negative COR values when incorporating appendage and telson motion occurred because the appendage moved faster than the telson – and in the same direction as the telson – post-contact. This is somewhat equivalent to penetration of a target, but in the mantis shrimp case, the appendage continued to rotate past the point of contact with the telson, as the telson rotated out of the way. My measurements of COR that incorporated appendage movement only (i.e. Eqn 1) did not allow for negative values of COR because they did not account for the direction of appendage movement (following methods in Taylor and Patek, 2010). However, in most (21/28) strikes, the appendage continued to move in the same direction as it did pre-contact. In live interactions like those studied here, it seems more accurate to account for the directionality of both appendage and telson movement when calculating COR, i.e. to calculate COR using Eqn 2. However, some aspects important to this approach were beyond the scope of the present study. For example, analyses of two-particle impacts are improved by incorporating the angle of their collision (Hibbeler, 1992). While I measured the angle of appendage displacement at contact, I could not accurately measure the angle of contact between the appendage against the telson. Future work, for example using 3D videography approaches or mounting dead animals in coiled postures and conducting ball-drop tests from multiple angles (e.g. Chau et al., 2002; Cross, 2002), might better model telson–appendage impacts.

The fact that, in most cases, the appendage continued to move in the same direction as the telson after contact is relevant to our understanding of mantis shrimp strike biomechanics. This post-contact movement suggests that the appendage may still be powered after the strike is released. This power could come from continued uncoiling of the appendage exoskeletal spring, but could also come from continued muscle contraction after contact. While mantis shrimp have been shown to vary strike kinematics according to behavioral context (fighting versus feeding; Green et al., 2019) and the medium in which they strike (water versus air; Feller et al., 2020), prior work in muscle physiology has suggested that kinematic variation is driven solely by variable muscle activation before the strike is released and has not looked for muscle activation after strike release (e.g. Kagaya and Patek, 2016). Future work might look for muscle activity later in the strike motion, testing whether individuals ‘punch through’ a target.

Different COR measurement techniques resulted in different predictors of COR. When measuring COR from appendage motion only (Eqn 1), COR increased with increasing striking body mass (Fig. 5). When measuring COR from combined appendage and telson motion (Eqn 2), COR decreased with increasing appendage velocity at contact (Fig. 6). In the fields of engineering and sports science, there is often either no relationship or a negative relationship between object mass and COR (Asteriou and Tsiambaos, 2018; Chau et al., 2002; Haron and Ismail, 2012), contrasting my results (Fig. 5), while impact velocity and COR are negatively correlated (Asteriou and Tsiambaos, 2018), matching my results (Fig. 6).

The positive relationship between striking body mass and COR could simply reflect the greater amount of energy delivered by strikes from larger individuals, i.e. those with larger striking body masses. A prior study found that larger mantis shrimp deliver a greater amount of energy in their strikes (Green et al., 2019); my results (Fig. 5) suggest that in these high-energy strikes, a lower proportion of strike energy is dissipated by the telson. The direction of the appendage post-contact also affected the relationship between striking body mass and COR (Fig. 5). In strikes where the appendage continued to move past the point of telson contact, COR was generally higher for a given striking body mass, as compared with strikes where the appendage rebounded, moving back toward the striking individual after contact. Put another way, strikes that resulted in the appendage rebounding after contact had proportionally more energy dissipated. It is unclear what determines whether or not a strike rebounds, but this may be related to where on the telson a strike lands. Though I could not quantify strike location precisely, strikes that landed on the central carina of the telson were more likely to rebound (P.A.G., personal observation). Future work could use 3D videography or other techniques to quantify precisely where on the telson strikes land and relate this to the likelihood of rebounding, and resulting variation in COR.

Regarding the negative relationship between appendage velocity at contact and COR, one interpretation is that striking individuals increase strike velocity to increase the energy their opponents must dissipate when receiving strikes. If strike energy dissipation is an active process by strike receivers – for example, if energy dissipation requires abdominal muscle activation – then dissipating more energy from higher-velocity strikes might lead to more rapid exhaustion by strike receivers, leading to a more rapid retreat. This hypothesis could be tested by combining high-speed video approaches like those used here with measurements of muscle physiology or metabolism after contests. In hermit crabs, competitors that received more powerful impacts from their opponent's shell (as measured by sound intensity) had decreased hemolymph glucose concentrations and were more likely to lose the contest (Briffa and Elwood, 2002); however, these physiological metrics were not linked to impact kinematics per se.

It was somewhat surprising that no other factors I analyzed affected COR. For example, I hypothesized that strike receivers that coiled their telson more tightly might dissipate more strike energy, but the degree to which the telson was coiled did not predict COR. It could be that, once the telson is lifted off the substrate and the body can flex upon receiving a strike, changing the degree of telson coil has minimal importance. Indeed, although energy dissipation varied alongside striking body mass (Fig. 5) and velocity at contact (Fig. 6), at minimum, mantis shrimp dissipated ∼75% of strike energy. Variation greater than this already-high value may have little importance, leading to relaxed selection on variation in the telson coil behavior. An alternative hypothesis for why individuals vary in the degree of telson coil relates to contest behaviors. Individuals outside their burrows frequently coil their telsons tightly in front of their bodies (Fig. 1); this allows them to both receive strikes safely and rapidly uncoil to deliver strikes in return (Green and Patek, 2018). By contrast, individuals already in their burrows often simply hold their telsons outside the burrow to receive strikes without tightly coiling them, receiving multiple strikes without delivering strikes themselves (P.A.G., personal observation). Coiling may therefore have evolved as a behavioral strategy for moving quickly from defense to attack, as opposed to, or in addition to, a mechanism of increasing energy dissipation. Furthermore, in natural contests, individuals inside burrows can sometimes wedge their telsons against the side of the burrow, which may change energy dissipation dynamics in ways that were not accounted for here.

Finally, Taylor and Patek (2010) found that larger mantis shrimp (measured by total body mass) had telsons that dissipated more strike energy (i.e. lower COR values). While I initially attempted to include the body mass of individuals receiving strikes in my models predicting COR, I ultimately removed this variable because it was highly collinear with the mass of the striking individual's striking body (Pearson correlation=0.91). However, in follow-up models, I replaced appendage striking body mass with the body size (total body mass) of the individual receiving the strike. In these models, I found no effect of size on COR (Tables S5 and S6). The key difference between my results and those of Taylor and Patek (2010) is that I matched opponents for body size. This was done to encourage individuals to strike, as size-matched opponents exchange more strikes (Green and Patek, 2018). As a result of this size matching, the relative mass of the striking object (the appendage striking body) and the target (strike receiver body) were somewhat similar across strikes and individuals. In comparison, Taylor and Patek (2010) kept the mass of the striking object – a 1.02 g steel ball – consistent, while varying the mass of the target. Therefore, Taylor and Patek (2010) achieved greater variation in the relative object mass to target mass [coefficient of variation (CV) of Taylor and Patek (2010) object to target mass=72.1; CV of present study object to target mass=15.3). To better test whether COR scales with body size in natural contests, future work could less closely match the size of sparring competitors, or could use physical modeling approaches in which coiling is allowed but the relative mass of the striking object and the struck object are allowed to vary to a greater extent than in the present study.

Both classic (Garland and Losos, 1994; Lauder, 1995) and recent studies (Bauer et al., 2020; Green et al., 2021) in integrative organismal biology have discussed the importance of couching biomechanics in greater ecological or behavioral realism. Using engineering-based techniques to analyze impacts from freely behaving competitors, my data suggest that both morphology and behavior – how morphology is used (Garland and Losos, 1994) – contribute to impact resistance in mantis shrimp. Given the widespread nature of impacts in contexts from foraging (Spagna et al., 2009; Vailati et al., 2012) to competition (Elwood and Briffa, 2001; Muñoz et al., 2012), and the importance of impact resistance to survival and reproduction, incorporating analyses of morphology and behavior to understand impact energy dissipation could help us understand how impacts act as a selective force on organismal structure and function.

## Supplementary Material

10.1242/jexbio.247063_sup1 Supplementary information
