# Supplementary material for: Behavior and morphology combine to influence energy dissipation in mantis shrimp (Stomatopoda)
Source: J Exp Biol. 2024 May 9;227(9):jeb247063. doi: 10.1242/jeb.247063 (PMC11128283; doi:10.1242/jeb.247063)
Supplement: Supplementary information [file jexbio-227-247063-s1.pdf]

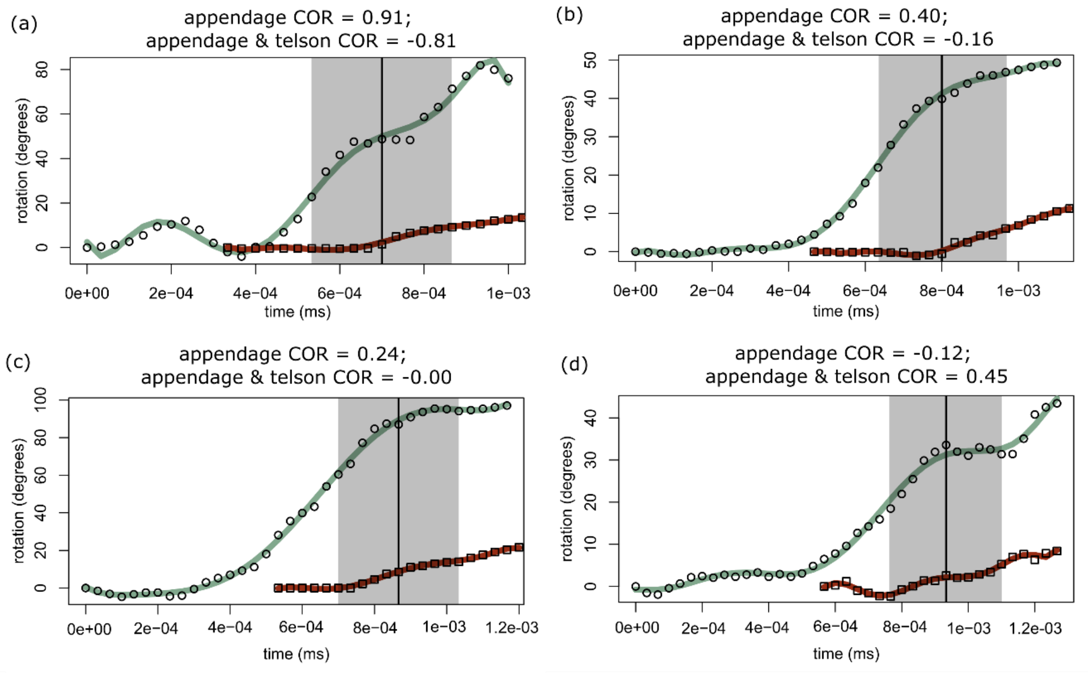

Fig. S1. Examples of rotation (degrees, y-axis) as a function of time (milliseconds, x-axis) for four strikes spanning variation in COR. Open circles and green line show raw data and polynomial fit, respectively, for appendage motion. Open squares and red line show raw data and polynomial fit, respectively, for telson motion. Titles of each plot show COR data for each strike.

Journal of Experimental Biology • Supplementary information

Table S1. Results of LMM predicting COR as measured by linear velocity from the fixed effect of dataset type. Table lists overall effect of dataset type, as well as post-hoc tests of individual contrasts. Dataset type is: "T&P 2010", referring to data from Taylor and Patek (2010); "Present (app)", referring to data from present study as measured by appendage motion only; and "Present (app + telson)", referring to data from present study as measured by both appendage and telson motion. Significant P-values (P < 0.05) are indicated by italics.

Fixed effect		β	SE	Test statistic*	P
Intercept				111.40 (χ^2)	*< 0.01*
Dataset	Overall effect			63.22 (χ^2)	*< 0.01*
Contrasts	T&P 2010 : Present (app)	0.25	0.07	3.53 (t)	*< 0.01*
	T&P 2010 : Present (app + telson)	0.59	0.08	7.41 (t)	*< 0.01*
	Present (app) : Present (app + telson)	0.34	0.06	5.44 (t)	*< 0.01*

Random effect	Variance	SD
Strike receiver ID	0.00	0.07
Striking individual ID	0.00	0.00

* Test statistic column lists test statistic value and relevant test statistic, either Chi-squared or t-value.

Table S2. Results of LMM predicting COR as measured by linear velocity of appendage motion only. Significant P-values (P < 0.05) are indicated by italics.

Fixed effect		B	SE	χ^2 (df)	P
Intercept		0.06	0.05	1.68 (1)	0.20
Velocity at contact		-0.00	0.02	0.01 (1)	0.93
Contact angle		-0.04	0.03	1.74 (1)	0.19
Appendage direction post-contact	Rebound	0.00	0.00		
	Continue	0.29	0.05	30.97	*<0.01*
Coil angle		-0.02	0.03	0.29 (1)	0.59
Striking body mass		0.11	0.03	11.01 (1)	*<0.01*
Glancing blow	N	0.00	0.00		
	Y	0.02	0.05	0.08 (1)	0.77
Velocity at contact : striking body mass		-0.02	0.03	0.52 (1)	0.47

Random effect	Variance	SD
Strike receiver ID	0.00	0.06
Striking individual ID	0.00	0.10

Table S3. Results of LMM predicting COR as measured by linear velocity of both telson and appendage motion. Significant P-values (P < 0.05) are indicated by italics.

Fixed effect		B	SE	χ^2 (df)	P
Intercept		-0.03	0.08	0.13 (1)	0.72
Velocity at contact		-0.22	0.08	8.16 (1)	*<0.01*
Contact angle		0.01	0.08	0.01 (1)	0.93
Coil angle		-0.00	0.08	0.00 (1)	0.97
Striking body mass		-0.04	0.08	0.23 (1)	0.63
Glancing blow	N	0.00	0.00		
	Y	0.03	0.13	0.06 (1)	0.80
Velocity at contact : striking body mass		-0.00	0.11	0.00 (1)	0.99

Random effect	Variance	SD
Strike receiver ID	0.00	0.00
Striking individual ID	0.00	0.00

Table S4. Results of LMM predicting COR, as measured by both telson and appendage motion, and in which contact frame was altered in four strikes such that velocity of contact was greater than zero.

Fixed effect		β	SE	χ^2 (df)	P
Intercept		-0.01	0.08	0.01 (1)	0.93
Velocity at contact		-0.12	0.10	1.47 (1)	0.23
Contact angle		-0.00	0.07	0.00 (1)	0.96
Coil angle		-0.04	0.07	0.27 (1)	0.60
Striking body mass		-0.04	0.07	0.43 (1)	0.51
Glancing blow	N	0.00	0.00		
	Y	0.10	0.12	0.78 (1)	0.38
Velocity at contact : striking body mass		0.05	0.09	0.34 (1)	0.56

Random effect	Variance	SD
Strike receiver ID	0.00	0.00
Striking individual ID	0.02	0.13

Table S5. Results of LMM predicting COR, as measured by both telson and appendage motion, and in which striking body mass is replaced by the size (mass) of the struck individual.

Fixed effect		β	SE	χ^2 (df)	P
Intercept		-0.07	0.10	0.51 (1)	0.48
Velocity at contact		-0.32	0.09	12.30 (1)	*< 0.01*
Contact angle		-0.11	0.13	0.68 (1)	0.41
Coil angle		0.07	0.14	0.68 (1)	0.41
Body mass of struck individual		-0.03	0.07	0.13 (1)	0.72
Glancing blow	N	0.00	0.00		
	Y	0.25	0.22	1.34 (1)	0.25
Velocity at contact : striking body mass		-0.06	0.15	0.13 (1)	0.71

Random effect	Variance	SD
Strike receiver ID	0.00	0.00
Striking individual ID	0.00	0.00

Table S6. Results of LMM predicting COR, as measured by appendage motion only, and in which striking body mass is replaced by the size (mass) of the struck individual.

Fixed effect		B	SE	χ^2 (df)	P
Intercept		0.06	0.07	0.73 (1)	0.39
Velocity at contact		-0.01	0.03	0.25 (1)	0.62
Contact angle		-0.02	0.04	0.22 (1)	0.64
Appendage direction post-contact	Rebound	0.00	0.00		
	Continue	0.28	0.06	20.98	*<0.01*
Coil angle		-0.03	0.04	0.49	0.48
Body mass of struck individual		0.10	0.05	3.79 (1)	0.05
Glancing blow	N	0.00	0.00		
	Y	0.00	0.06	0.01 (1)	0.95
Velocity at contact : striking body mass		-0.03	0.04	0.60 (1)	0.44

Random effect	Variance	SD
Strike receiver ID	0.01	0.11
Striking individual ID	0.00	0.10

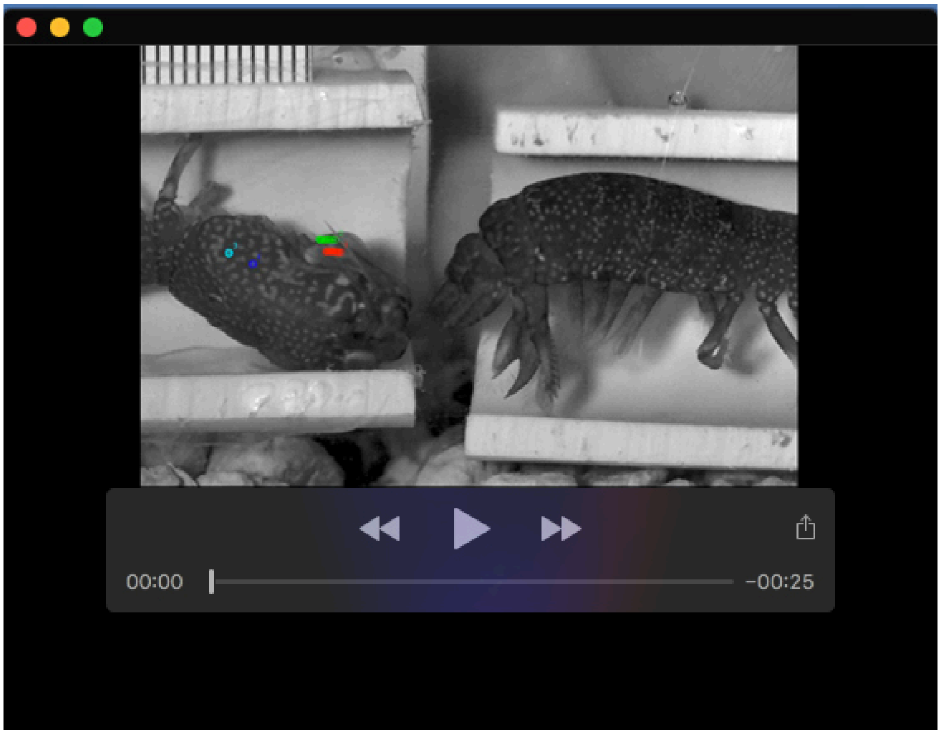

Movie 1. Three examples of digitized strike videos, highlighting the movement of the telson and body after receiving a strike. This whole-body movement likely contributes to lower COR values (higher energy dissipation) when measured from freely-interacting animals, as compared to focusing solely on morphology. In each video, colored points show landmarks digitized during experiment and from which displacement data were obtained. Frame rates in each video were 30,000 frames/second, played back at 7 frames/second. The third video shows an example of a glancing blow.
